# A One-Year Study Using Digital Biomarkers From Sensing Technologies to Assess Changes in Physical Activity Levels and Sleep Quality in Nursing Home Residents With Dementia: Observational Study

**DOI:** 10.2196/95194

**Published:** 2026-08-28

**Authors:** Lydia D Boyle, Monica Patrascu, Bettina S Husebo, Kristoffer Haugarvoll, Ole Martin Steihaug, Brice Marty

**Affiliations:** 1 Center for Elderly and Nursing Home Medicine, University of Bergen, Årstadveien 17, Bergen, Vestland, 5009, Norway, 47 040518081; 2 Neuro SysMed, Haukeland University Hospital, Bergen, Vestland, Norway; 3 Haraldsplass Diakonale Sykehus, Bergen, Vestland, Norway

**Keywords:** sensors, sensing technology, wearables, sleep disturbance, physical activity, nursing homes, dementia

## Abstract

**Background:**

Proxy-rated questionnaires remain the standard for the assessment of activity and sleep for people with dementia living in nursing homes. Sensing technologies, such as wearables, can generate continuous data that provide quantitative insights into daily activities and behavioral and psychological symptoms, such as sleep disturbance. This study explores the use of sensing technologies in the detection of changes in physical activity levels and sleep behaviors over time.

**Objective:**

This study aims to explore the long-term capabilities of multimodal sensing technologies for assessing physical activity levels and sleep quality using selected digital biomarkers for nursing home residents with dementia. Objectives were for observation to be aligned with real-world conditions in which such sensing technologies would be applied within a nursing home environment and to assess whether distinct differences in selected digital biomarkers can be observed accurately and reliably longitudinally.

**Methods:**

This study included 11 participants (79‐93 y) recruited from 2 dementia care units in Norway. A smartwatch (Garmin Vivoactive 5 or Garmin Venu 3) and a radar-based system (Vital Things, Somnofy) were used to collect 7 days and 6 nights of data on physical activity levels and sleep quality at baseline, 6 months, and 1 year. The Personal Self-Maintenance Score and Neuropsychiatric Inventory–Nursing Home Version (nighttime behaviors section K) were also administered. Digital biomarkers included Euclidean norm minus one (ENMO), sleep efficiency (SE), wake after sleep onset (WASO), sleep regulatory index (SRI), sleep fragmentation index (SFI), total sleep time (TST), and time out of bed (no presence).

**Results:**

A total of 9 participants were included in the final analysis. Differences were found in nighttime ENMO (*P*=.01) and in 4 of the sleep biomarkers: TST (*P*=.02), SE (*P*=.02), WASO (*P*=.01), and SRI (*P*=.01). The long-term reliability of the group ENMO was poor (intraclass correlation coefficient 0.00‐0.02); however, it was moderate to strong (0.58‐0.79) between individuals at each time point. The adherence and acceptability of the technologies was high (88%-96%), and the application of the devices was well tolerated by the participants, with no adverse events.

**Conclusions:**

The use of sensing technologies could enable more objective, data-driven future care models for people with dementia residing in nursing homes; however, the results emphasized in this study require the recommendation for cautious, well-designed use of digital biomarkers for clinical decision-making.

## Introduction

### Background

The global prevalence of dementia is expected to reach 139 million by 2050 [1], and in countries such as Norway, more than two-thirds of nursing home residents live with dementia [2]. Monitoring neuropsychiatric symptoms (NPSs) and changes in physical activity typically relies on proxy-rated assessments, which are limited by subjectivity, retrospective bias, and low sensitivity to subtle or short-term change [3]. As health care systems face increasing workforce shortages, there is a growing need for scalable, objective approaches that can support continuous monitoring and individualized care [4]. Recent advances in sensing technologies, including wrist-worn wearables and contactless radar systems, offer minimally obtrusive, real-time data collection in naturalistic environments and may help address these challenges.

Physical inactivity is widespread in nursing home environments [5,6], with daily routines dominated by sedentary behaviors such as sitting, resting, and sleeping [7]. Despite the importance of physical activity for maintaining function, there are no standardized recommendations for activity dosage in people with advanced dementia [8]. Although the relationship between daytime activity and sleep quality remains insufficiently understood, several studies suggest that higher daytime activity may be associated with improved sleep [9,10]. Functional ability is typically assessed using proxy-rated activities of daily living (ADL) questionnaires, but these tools are retrospective and infrequently administered, reducing their use for detecting early or subtle decline, which is an important limitation given the strong association between functional loss and increased morbidity and mortality [11].

Accelerometry provides several methods for quantifying physical activity, including step counts, intensity, frequency, and vector magnitude [12], and common metrics derived from raw acceleration data include Euclidean norm minus one (ENMO), mean amplitude deviation (MAD), Monitor-Independent Movement Summary (MIMS), activity index, and Rate of Change Acceleration Movement (ROCAM) [12,13]. A recent feasibility study in nursing home residents with dementia demonstrated that commercial wearables could accurately classify physical activity over a 24-hour period [14], supporting the promising potential of sensing technologies in dementia care [15,16]. However, research validating activity classification and intensity thresholds in older adults with dementia remains limited, as demonstrated in a systematic review by Gorman et al [13] exploring physical activity and sedentary behaviors in older adults, finding that there are no standardized accelerometry-based thresholds for moderate-to-vigorous or sedentary activity, with most cut-offs validated in healthy adults rather than cognitively impaired populations. Research using wrist-worn accelerometers and actigraphy in a cohort of 415 nursing home residents in Australia demonstrated that these devices could capture patterns of inactivity and sleep duration in this population. The study also highlighted practical challenges, particularly around device accuracy and participant adherence, which was particularly low (<50%), concluding that additional work is needed to refine sensor placement, device characteristics, and methodological approaches for use in a nursing home setting [7].

Sleep disturbances are prevalent in nursing home residents and affect up to 90% of those with dementia [17], with common issues including insomnia, sundowning, restless legs, and fragmented sleep. Sleep quality is particularly compromised in people with dementia residing in nursing homes [18,19], and although nonpharmacological interventions are recommended [20], psychotropic medications remain widely used and may worsen sleep or increase the risk of injury [21], contributing to further NPSs such as agitation, anxiety, and nighttime restlessness [18]. Key sleep metrics, including efficiency, latency, wakefulness after sleep onset, fragmentation, regulation, and total sleep, decline with age and are further impaired for people with dementia [22]. Autonomic regulation of sleep also changes with age and neurodegeneration, particularly regarding parasympathetic nervous system involvement in the dampening of the sympathetic nervous system during deep sleep [23]. Sleep durations of 6 to 9 hours have been associated with improved parasympathetic function in older adults with neurodegenerative disease [24].

Polysomnography (PSG) remains the gold standard for sleep assessment but is often impractical and burdensome for people with dementia in nursing homes [25]. Consumer wearables provide sleep estimates using accelerometry and photoplethysmography, but they frequently overestimate sleep in individuals with sleep dysfunction [26] and vary in accuracy [27]. Newer commercial wearable models offer sleep staging, and although sleep sensitivity tends to be adequate, the poor detection of awake periods reduces accuracy, making these devices not yet adequate for research or clinical standards and not validated in an older adult population [28]. Radar-based systems [29] offer a contact-free alternative capable of monitoring sleep in free-living environments. These systems produce digital biomarkers comparable to PSG and show promising accuracy [30], though further research is needed to confirm their reliability and clinical use for longitudinal monitoring in real-world dementia care settings.

### Study Aim and Objectives

This study aims to explore the long-term capabilities of multimodal sensing technologies for assessing physical activity levels and sleep quality using selected digital biomarkers for nursing home residents with dementia. Objectives were for observation to be aligned with real-world conditions in which such sensing technologies would be applied within a nursing home environment, and to assess whether distinct differences in selected digital biomarkers can be observed accurately and reliably longitudinally.

## Methods

### Design and Setting

This study is related to an observational pilot study: digital phenotyping for changes in activity at the end of life in people with dementia (DIPH.DEM): an observational trial based on sensing technology. DIPH.DEM is a cornerstone study for a larger observational study titled “Decoding Death and Dying in People With Dementia by Digital Thanotyping” (5-D; REK 657596, NEM 2023/166). DIPH.DEM was designed to improve the overall quality, recruitment strategies, and efficacy of the larger 5-D study. This paper is an extension of a previous article [31], which used the DIPH.DEM baseline data (description in Table 1) to investigate the association between daytime activity and sleep quality in people with dementia residing in a nursing home. The DIPH.DEM cohort and study design have previously been described in detail in 2 previous articles [31,32].

Participants (n=11) 65 years or older were recruited from 2 dementia wards in a Norwegian nursing home. Eligibility for the study included that the participants had lived in the nursing home for more than 6 weeks and had a previous diagnosis of moderate-to-severe cognitive impairment or dementia, confirmed within the medical record and/or using the Clinical Dementia Rating (CDR) scale administered at baseline. Exclusion criteria included the presence of delirium at the baseline assessment and a current life expectancy of less than six weeks as suggested by the multidisciplinary nursing home team. Data were collected from February 2024 to May 2025. The proxy-rated questionnaires and continuous digital monitoring using sensing technologies, Garmin Vivoactive 5 or Garmin Venu 3 and Vital Things Somnofy, were conducted at 3 distinct time points: baseline, 6 months, and 1 year.

**Table 1.** Sample characteristics from the DIPH.DEM^a^ study (N=11) [31].

Demographics of study participants	Values
Age (y), mean (SD; range) (n=11)	84.0 (6.1; 77‐93)
Gender, n	
Female	8
Male	3
Months living in the nursing home, mean (SD; range) (n=11)	17.6 (12.5; 3‐42)
Dementia as primary diagnosis, n	6
Alzheimer dementia, n	3
Parkinson disease, n	1
Clinical Dementia Rating score, mean (SD; range) (n=11)	2.2 (0.7; 1‐3)
Mild, n	4
Moderate, n	5
Severe, n	2
Personal Self Maintenance Score, mean (SD; range) (n=11)	15.7 (5.7; 8‐23)
Comorbidities (GMHR^b^), mean (SD; range) (n=11)	2.6 (0.7; 1‐3)
Neuropsychiatric Inventory (NPI-NH^c^)–12 subsections, mean (SD; range)	
Agitation (n=5)	2.2 (2.2; 1‐6)
Delusion (n=4)	3.0 (2.4; 1‐6)
Hallucinations (n=3)	3.0 (2.6; 1‐6)
Depression (n=4)	2.0 (1.4; 1‐4)
Anxiety (n=3)	1.3 (0.6; 1‐2)
Apathy (n=4)	5.8 (4.6; 1‐12)
Irritability (n=5)	3.6 (2.9; 1‐8)
Euphoria (n=1)	8.0 (0.0; 8‐8)
Disinhibition (n=4)	1.5 (0.6; 1‐2)
Aberrant motor behavior (n=4)	1.0 (0.0; 1‐1)
Nighttime behavior disturbances (n=6)	4.7 (2.9; 1‐8)
Appetite or eating (n=4)	4.0 (2.9; 1‐8)
Total medication use, mean (SD; range) (n=11)	7.6 (3.4; 2‐13)
Psychotropic drug use, mean (SD; range) (n=8)	1.9 (1.4; 1‐5)

a DIPH.DEM: digital phenotyping for changes in activity at the end of life in people with dementia.

b GMHR: General Medical Health Rating.

c NPI-NH: Neuropsychiatric Inventory-Nursing Home.

### Ethical Considerations

Verbal and written informed consent were obtained in direct conversation with the residents who demonstrated sufficient capacity to consent. For those lacking this ability, we obtained consent from the resident’s legal guardian, usually a family member, after explaining the aims and protocol of the study. The DIPH.DEM study was approved by the Regional Committee for Medical and Health Research Ethics (REK) in Norway in October 2023 (approval number 634938).

### Traditional Questionnaires

Traditional outcome measures used in this study were the Neuropsychiatric Inventory–Nursing Home version (NPI-NH) [33], assessing symptoms of sleep disturbance (Nighttime Behaviours section K), and the Physical Self Maintenance Scale (PSMS) [34], assessing functional activity levels and adverse effects of inactivity. The NPI-NH (section K) [35] includes questions about difficulty falling asleep, frequent nightly awakenings, wandering, dressing in the middle of the night, and waking earlier than normal to begin the day. The PSMS [36] includes functional activities of ambulation, bathing, grooming, dressing, toileting, and feeding. To define the severity and level of disturbances within this study, we chose to use a global scoring system from 6 to 30 (6 questions and 5 possible answers), with lower scores indicating lower dependency and higher scores indicating higher dependency.

Additional baseline measurements reported in this study were the General Medical Health Rating (GMHR) [37] for the assessment of general health and comorbidities, 4AT (Assessment Test for delirium) [38], and the CDR [39]. The CDR questionnaire (range 0‐3) is a widely accepted global scale developed to clinically identify the presence of dementia and stage its severity (score of >0.5: cognitive impairment/likely dementia; 1: mild; 2: moderate; 3: severe dementia). Within our study, participants were included in the study with a score equal to or greater than 1 on the CDR. The GMHR (range 1‐4) assesses the number and impact of comorbidities (1=very good; 2=good; 3=moderate, and 4=bad health). The 4AT Test (range 0‐12) is a screening tool for delirium and consists of 4 questions evaluating alertness, cognitive function, and acute change, with a score of 4 or above indicating potential delirium. All traditional questionnaires used were previously validated and provided in the Norwegian language.

### Sensing Technologies and Digital Biomarkers

The sensing technologies used in the study were Somnofy (Vital Things AS, Trondheim, Norway) [40] version 0.7 with sleep algorithm 1.0 and Garmin (Garmin Ltd., Switzerland) Vivoactive5 smartwatch [26] firmware version 11.14 or Garmin Venu 3 smartwatch (X) firmware version 12.11. Digital biomarkers used in this study were acceleration-based physical activity estimates using ENMO, sleep efficiency (SE), wake after sleep onset (WASO), sleep regulatory index (SRI), total sleep time (TST), sleep fragmentation index (SFI), and no presence (time out of bed) (Table 1). Garmin Vivoactive5 was used for data collection at baseline and 6 months and upgraded to Garmin Venu3 at the 1-year time point for improved battery capacity and data storage capabilities. Both smartwatch models contained accelerometers used to capture raw acceleration signals. The Venu3 includes additional sensors, a barometric altimeter, gyroscope, and the updated Elevate V5 heart-rate sensor, while the earlier model uses the V4 sensor. Only accelerometer-derived raw data were analyzed in this study. Participants wore the device continuously for 7 days at each data collection point (baseline, 6 months, 1 year), including during sleep and bathing.

Garmin wearables, including Vivoactive and Venu models, demonstrate acceptable validity and strong interdevice reliability for step counting and movement assessment in laboratory and free-living settings [41,42]. Prior studies report accurate step detection across walking speeds and age groups, including older adults with slower gait patterns [43,44]. Garmin devices have also shown reliable estimation of physical activity using raw acceleration data [45], and ENMO-based thresholds applied to wrist-worn Garmin sensors provide high sensitivity and specificity for distinguishing sedentary and light-intensity behaviors [46].

### Data Extraction and Preprocessing

#### Physical Activity Levels: Euclidean Norm Minus One—Acceleration Data

Raw data parameters for the smartwatches were synced, paired, and retrieved using a hub through Fitrockr [47], a data broker based in Berlin, Germany, and acceleration was collected at 25 Hz. The extracted data were imported and converted in MATLAB and STATA-compatible types. Based on the Unix time timestamps to Unix time, the data were segmented into 14-hour periods (07:00:00 AM-08:59:59 PM) representing daytime; the first and last partial days were excluded to only consider full day recordings (24 h), resulting in 5 days and 6 nights of total acceleration data per participant. Nighttime physical activity was considered within the remaining timeframe of 09:00:00 PM-06:59:59 AM and based on nightly routines at the nursing home. The raw acceleration data were converted to the universal metric ENMO for physical activity analysis. ENMO is the vector magnitude of the 3 raw signals (*x*, *y*, *z*) minus 1. One-minute periods were used during daytime (07:00:00 AM-08:59:59 PM), nighttime (09:00:00 PM-06:59:59 AM), and daily 24-hour (07:00:00 AM-06:59:59 AM) measurements (mean, median, SD). The thresholds for physical activity levels using ENMO classifications were determined according to previous literature [46] and included sedentary activity to time (<18 milli-g), light physical activity (18‐22 milli-g), light-to-moderate activity (>22‐60 milli-g), and moderate-to-vigorous physical activity (MVPA; >60 milli-g). The calculation used for conversion of raw accelerometry data to ENMO (units: milli-g) was as follows:


$${ENMO}_{i}=\sqrt{x{\left(t\right)}_{i}^{2}+y{\left(t\right)}_{i}^{2}+\;z{\left(t\right)}_{i}^{2}\;}-1000\;\;\;$$

#### Digital Sleep Biomarkers

Sleep metrics (SE, SRI, WASO, time in no-presence, and SFI) were exported directly from the Somnofy platform. All Somnofy sleep data were manually reviewed to remove daytime sleep episodes occurring between 07:00 AM and 08:59 PM. These daytime events were documented separately to avoid influencing nighttime sleep estimates. Daytime sleep is not reported here, as the radar system only detects sleep occurring within its sensing range (ie, in bed) and therefore does not holistically capture daytime sleep, which may occur elsewhere than the bed. The nighttime window was selected to align with the routine administration of psychotropic medications for sleep disturbance (typically around 09:00 PM) and standard bedtime procedures at the nursing home. Participants were excluded if fewer than 10 hours of nighttime sleep data (one full night) were not available.

SE was calculated as the proportion of total sleep time relative to time in bed, expressed as a percentage from 0 to 100. SRI, also provided by Somnofy, reflects the consistency of sleep-wake patterns across days and requires at least 4 consecutive nights of data; values are expressed as a percentage and range from 0 to 100. WASO represents the cumulative minutes awake after initial sleep onset. The SFI score quantifies sleep fragmentation as the number of transitions from deep to light sleep per hour of sleep and is expressed as a percentage from 0 to 100. Somnofy additionally reports time in “no presence,” defined here as time or minutes spent outside the sensor’s effective range (>3 m), which was interpreted as time out of bed during the nighttime period. All wake periods overlapping with “no presence” were manually checked to confirm accuracy.

### Statistical Analysis

Descriptive statistics and narrative synthesis are provided including mean, median, minimum, maximum, and SD for all relative variables. The data were tested for normality and analyzed using MATLAB R2023a and STATA SE18.5; outliers were detected using box plots. The data were dependent, nonparametric, and not normally distributed resulting in the use of the Friedman test to assess differences between the 3 data collection time points. The Friedman test is used for one-way repeated measures ANOVA by ranks. A post hoc evaluation of the specific differences between the time points in which the null hypothesis was rejected was performed using the Wilcoxon signed-rank test. Kendall *W* interpretation of effect is as follows: 0.1‐0.29 small effect size, 0.3‐0.5 moderate effect size, and >0.5 large effect size. A sensitivity analysis was performed as a subanalysis of the significant results by removing influential extreme outliers, which were defined as totals beyond 1.5 times the IQR. The results were considered statistically significant at a *P*<.05, and at a *χ*^2^ (2 *df*) critical value of ≥5.991. Reliability as temporal stability is reported using an intraclass correlation coefficient (ICC 1,1 and ICC 3,1) defined with scores ranging from 0 to 1 and score interpretation: <0.5 as poor agreement, 0.5 to <0.75 as moderate agreement, 0.75 to <0.90 as good agreement, and 0.9 to 1.0 as excellent agreement.

## Results

Eleven participants were recruited from February 2024 to May 2025. Two participants passed away during the data collection period, resulting in final analysis of 9 participants. Data were missing for the following reasons: charging of the smartwatch (1‐2 hours on day 4) and periodic nonadherence from the participants (removal of the smartwatch for periods ≤2 hours). Adherence was defined as the total wear time (Garmin VivoActive5 or Venu3) calculated after the initial data extraction of the first and last day, and was 96% at baseline, 88% at 6 months, and 94% at 1 year. For the acceleration data, after the removal of partial days within the available data profiles (first and last day), there were a total of 135 acquisition periods available for the final analysis. Additionally, a total of 189 data acquisition periods, consisting of data from a total of 219 nights, were used for analysis of the sleep quality digital variables.

Participant characteristics and demographics at baseline for the DIPH.DEM study (n=11) can be found in Table 1. Participants were mostly female (8/11), age ranging from 77 to 93, living in the nursing home for a mean of 17.6 (SD 12.5) months, and with an average score of 2.2 on the CDR indicating a moderate level of dementia. Participants had a mean score of 2.6 (SD 0.7) on the GMHR, suggesting a moderate level of comorbidities consisting of greater than or equal to 3 chronic illnesses and concomitant medication use. Chronic diagnoses within the group included Alzheimer disease, Parkinson disease, diabetes, and heart disease. At baseline, assessed by the NPI-NH (section K for nighttime behavior), almost half (6/11) of the group was classified as having nighttime sleep behaviors defined within the study as frequent nighttime activity and awakenings. Total daily polypharmacy within the group ranged between 2 and 13 total medications per person, and the use of daily psychotropics due to sleep disturbances within the group profile ranged from 1 to 5 prescriptions per participant, per night.

**Figure 1.**
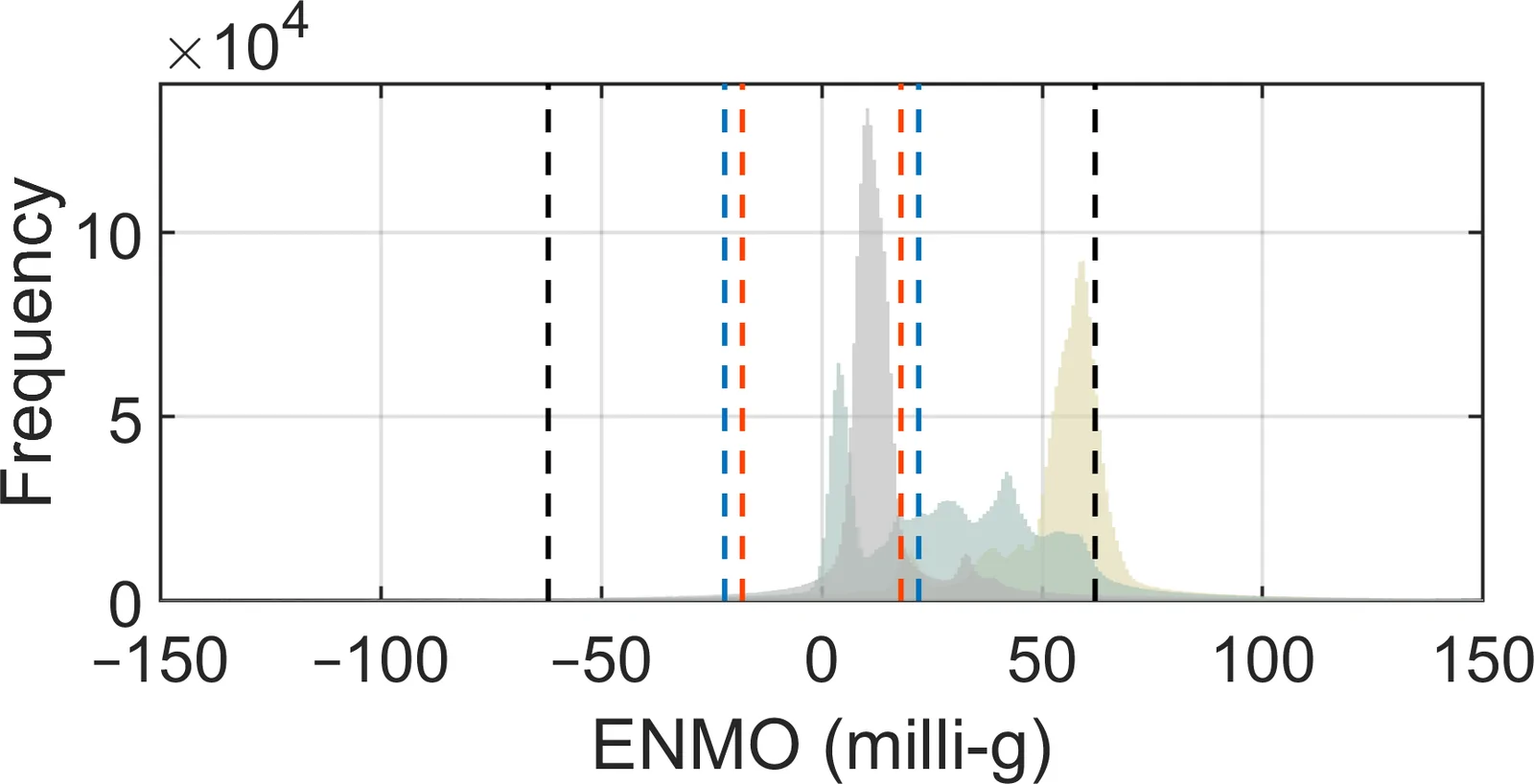
Distribution of 24-hour physical activity levels (Euclidean norm minus one [ENMO]) for one participant at baseline (yellow), 6 months (green), and 1 year (gray). Explanation of thresholds is as follows: space between orange lines=sedentary time (<18 milli-g), between red and blue lines=light physical activity (18‐22 milli-g), between blue and black lines=light-to-moderate physical activity (>22‐60 milli-g), all else from black lines=moderate-to-vigorous activity (>60 milli-g).

**Figure 2.**
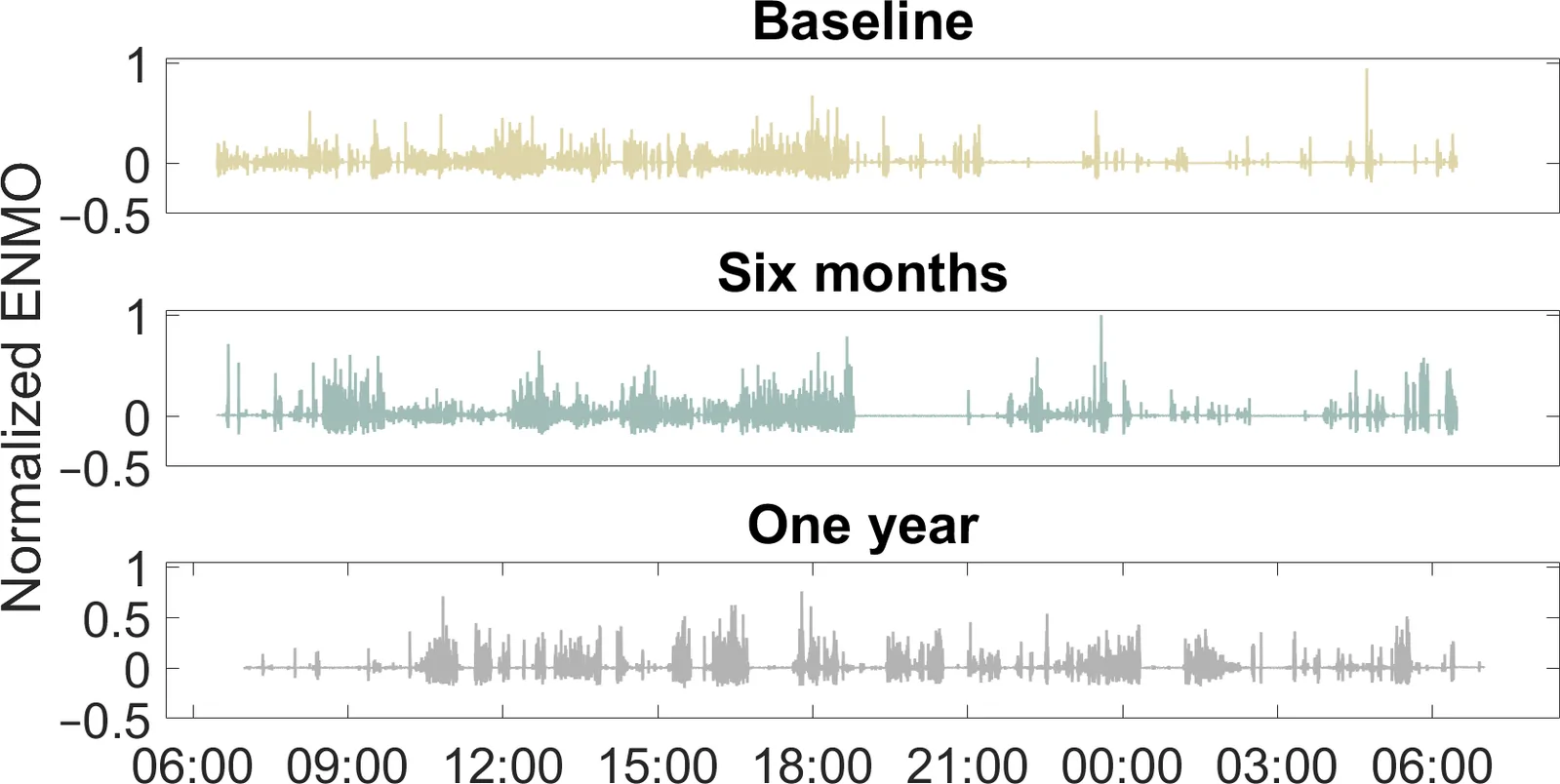
A 24-hour physical activity level. Definition of normalized Euclidean norm minus one (ENMO): the EMNO values were divided by the maximum value of the 3 days represented and are a snapshot for the same participant (Figure 1) at baseline, 6 months, and 1 year.

**Figure 3.**
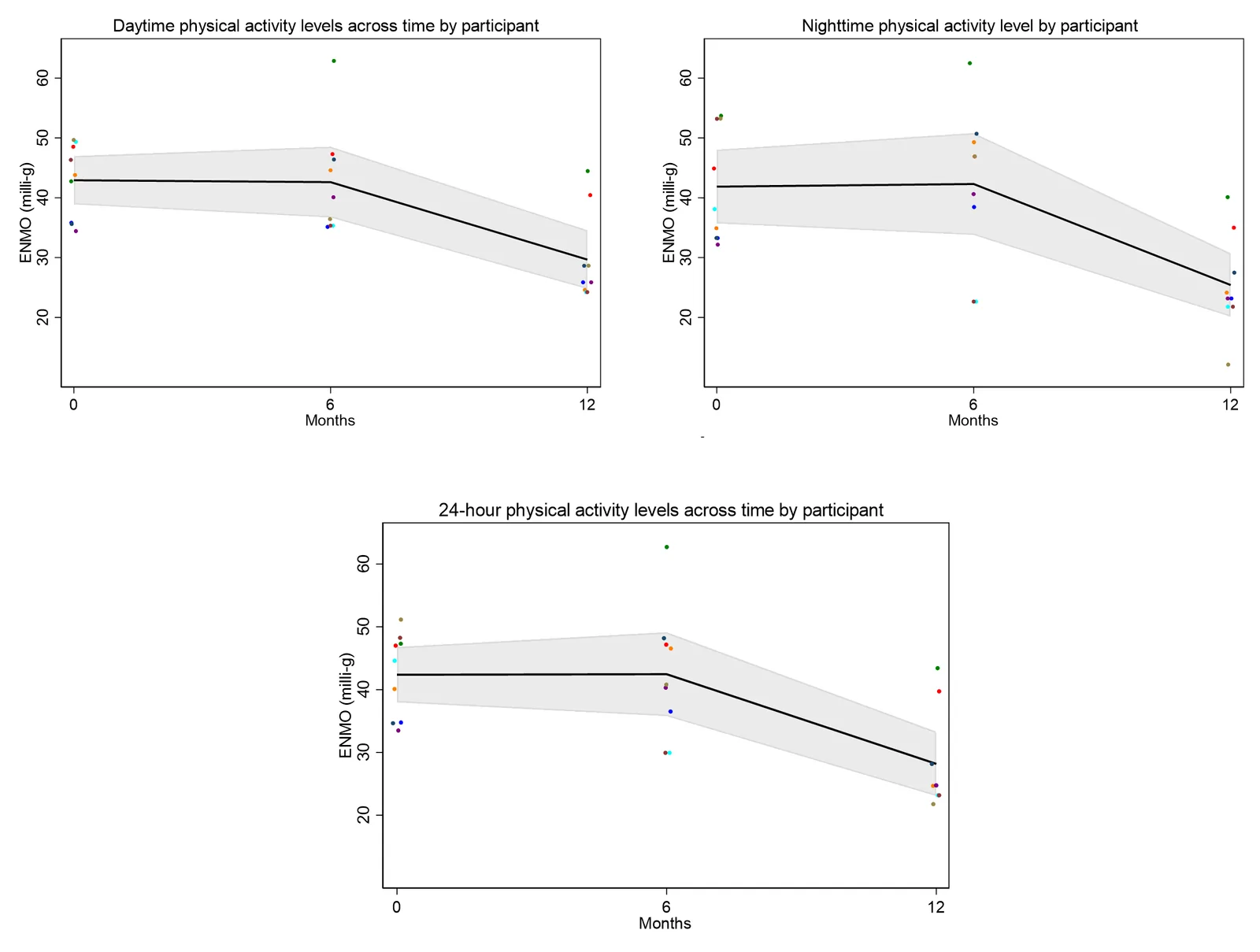
Mean differences of rank variance in Euclidean norm minus one (ENMO) representing physical activity levels, daytime, nighttime, 24 hours from baseline, 6 months, and 1 year. Each participant is represented by a different color “dot,” and the figures include all data points used for analysis for each participant. One “dot” then represents 1 data point. The gray band characterizes the IQR at 25/75%.

There were longitudinal differences in nighttime physical activity levels from baseline to 1 year (*P*=.01), with a declining trend (33% decrease from baseline to 1 year) in all ENMO means, most pronounced from 6 months to the 1-year time points (Figures 1–3). The majority of the group’s 24-hour activity intensities, as illustrated in Figure 1, were either sedentary threshold or below (<18 milli-g) or between sedentary time and light-to-moderate physical activity thresholds (18‐60 milli-g). In Figure 2, we observe a loss of day or nighttime rhythm or flattening of ENMO intensities, most distinct at the 1-year time point, indicating that daytime ENMO more closely resembled the nighttime ENMO intensities at the 1-year time point. The temporal stability (ICC) within and between individuals over time was poor (0.00‐0.02; Table 2), suggesting that the acceleration data (ENMO) was not a reliable measurement longitudinally; however, variance between-persons at a single time point ranged from moderate to good (0.58‐0.79), suggesting that ENMO can distinguish between individuals cross-sectionally in a stable and meaningful way. After removing outliers and aggregating repeated measurements, there was no statistically significant change in nighttime ENMO across time points; however, the effect size remained moderate, indicating a meaningful degree of within-participant change over time.

**Figure 4.**
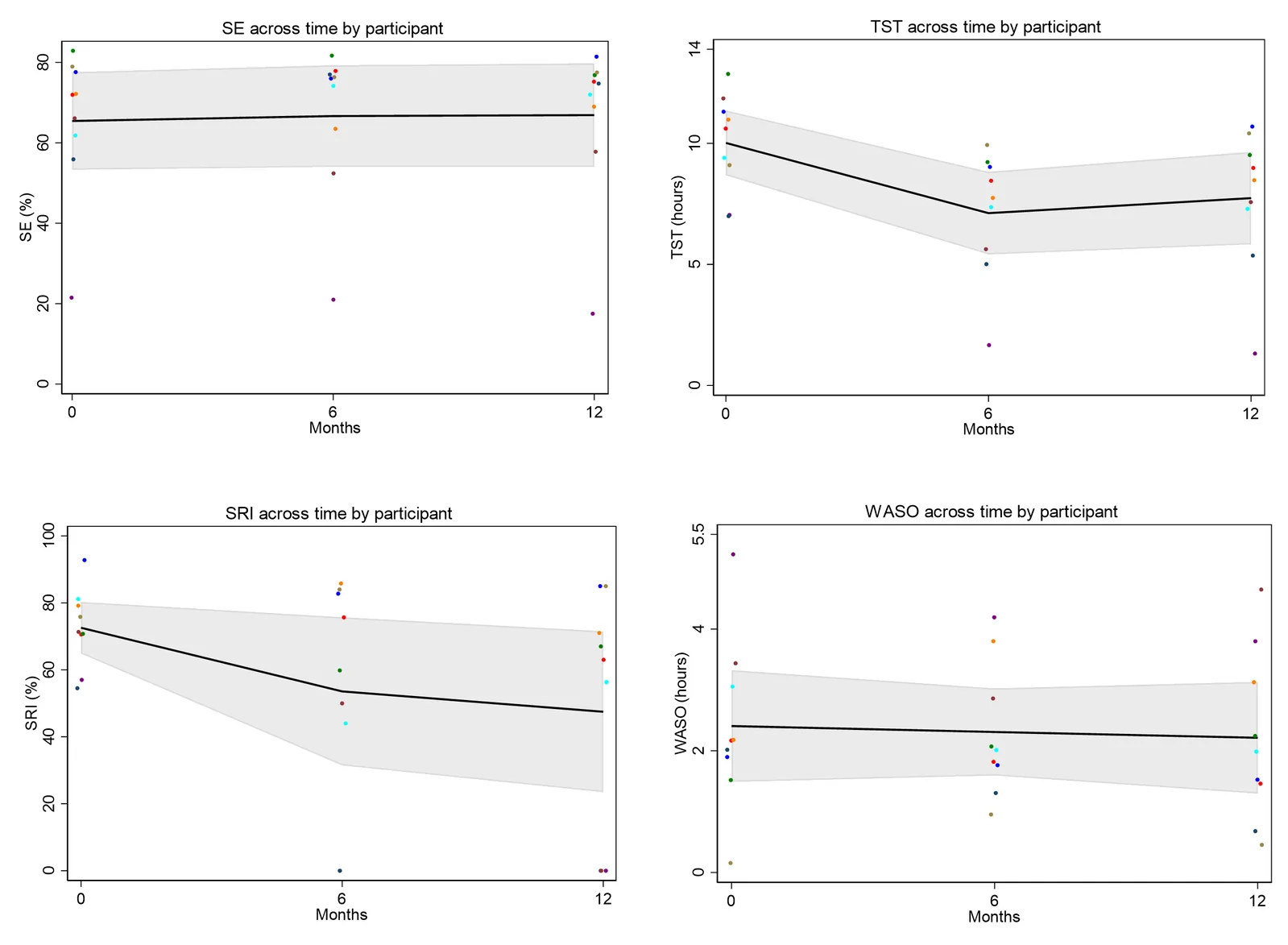
Median differences of rank variance in total sleep time (TST), wake after sleep onset (WASO), sleep efficiency (SE), and sleep regulatory index (SRI), Friedman test from baseline, 6 months, and 1 year. Each participant is represented by a different color “dot,” and the figures include all data points used for analysis for each participant. One “dot” then represents one data point. The gray band characterizes the IQR at 25/75%.

**Figure 5.**
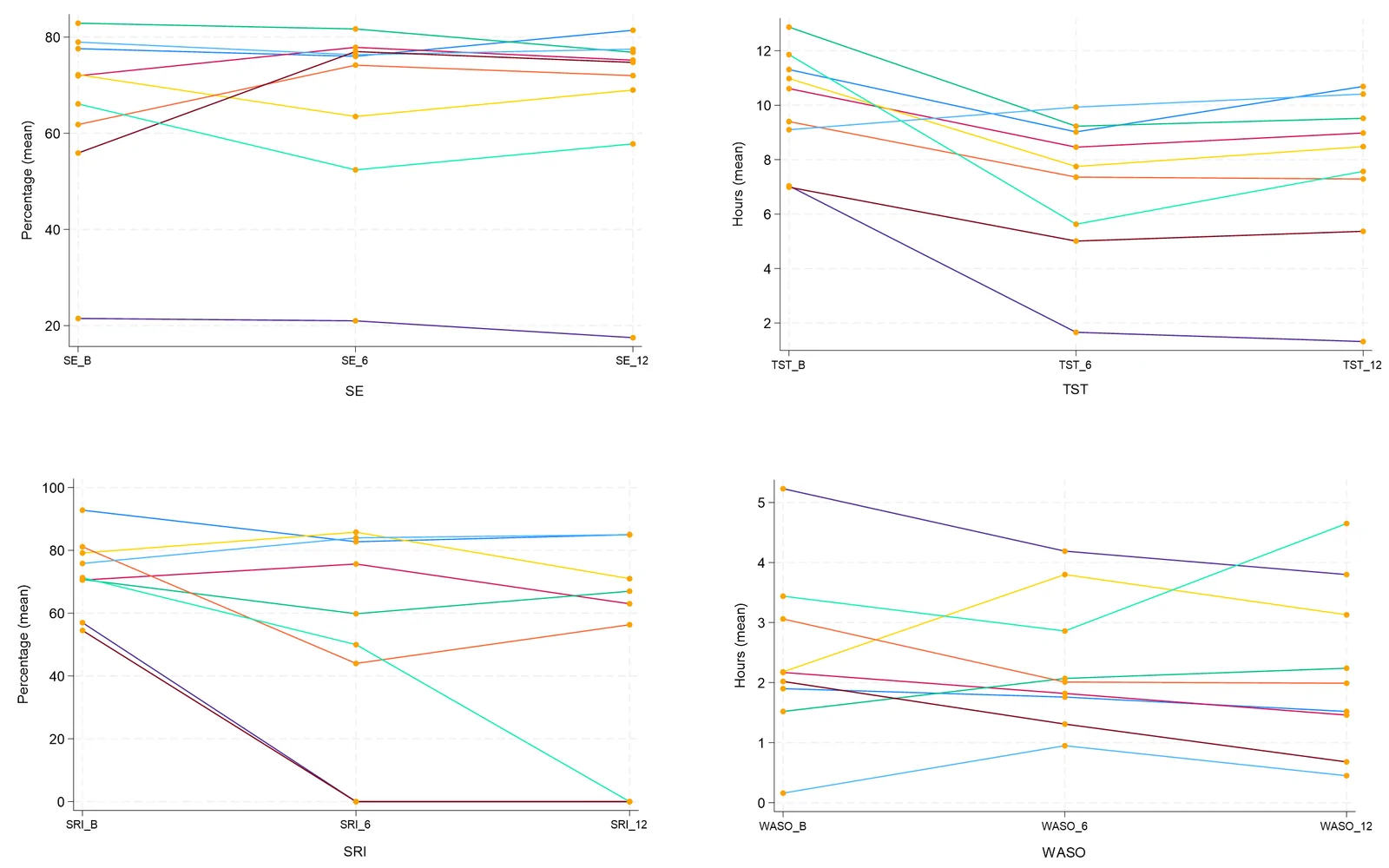
Longitudinal mean of individual trajectories at baseline (B), 6 months (6), and 1 year (12) for total sleep time (TST), wake after sleep onset (WASO), sleep efficiency (SE), and sleep regulatory index (SRI); each color represents a separate participant (n=9).

Four of the digital sleep quality biomarkers were found to have differences from baseline to 1 year: TST (*P*=.02), SE (*P*=.02), WASO (*P*=.01), and SRI (*P*=.01; Table 2, Figures 4 and 5). This suggests that these sleep quality digital biomarkers are sensitive enough over time to be used as a tool for identifying discrete changes in sleep quality. Temporal stability (ICC) of the sleep digital biomarkers over time was moderate to strong (0.54‐0.92; Table 2), with the most reliable longitudinal metrics being SE (0.92) and WASO (0.78). This indicates that these sleep biomarkers, derived from the Somnofy radar-based sensors, provided clinically meaningful data over time with minimal residual variance. A sensitivity analysis was performed for subanalysis of TST, SE, WASO, and SRI. After the removal of extreme outliers, SE and SRI showed no significant changes over time but moderate within-participant effect sizes. Both WASO and TST demonstrated longitudinal change, each with large effect sizes indicating substantial within-participant shifts.

**Table 2.** Longitudinal changes (baseline, 6 months, and 1 year) from selected sensing and traditional outcome measures (N=9)^a^.

Selected variables: longitudinal results at baseline, 6 months, and 1 year	Baseline		Six months		One year		Friedman test			Reliability
	Mean (SD)	Median (range)	Mean (SD)	Median (range)	Mean (SD)	Median (range)	Chi-square (*df*)	Kendall *W*	*P* value	ICC^b^
NPI-NH-K^c^ nighttime behaviors	5.4 (2.6)	6 (1‐8)	9 (0.0)	9 (9)	3 (2.4)	2.5 (1‐6)	11.0 (2)	0.46	.20	0.20
Personal Self Maintenance Scale—Physical Activity	17.3 (5.0)	17 (10‐23)	16.4 (4.6)	18 (8‐21)	17.8 (4.5)	20 (8‐22)	11.5 (2)	0.48	.18	0.46
Daytime activity (ENMO^d^) Garmin (milli-g)^e^ 07:00 AM-08:59 PM	42.9 (6.2)	43.8 (34.4‐50)	42.6 (9.1)	40.1 (35.1‐63)	29.7 (7.5)	25.9 (24.2‐45)	44.9 (2)	0.38	.24	0.01
Nighttime activity (ENMO) Garmin (milli-g) 09:00 PM-06:59 AM	41.9 (9.4)	38.1 (32.2‐54)	42.3 (13.0)	47.0 (22.7‐63)	25.4 (8.1)	23.2 (12‐40)	63.4 (2)	0.56	.01^f^	0.00
24-hour activity (ENMO) Garmin (milli-g) midnight-11:59 PM	42.4 (6.7)	44.6 (33.5‐51)	42.5 (10.2)	40.8 (30‐63)	28.2 (7.9)	24.7 (21.8‐43)	56.2 (2)	0.47	.05	0.02
Total sleep time (h)	10.0 (2.1)	10.6 (7‐13)	7.1 (2.6)	7.7 (2‐10)	7.7 (2.9)	8.5 (1‐11)	18.3^f^ (2)	0.76^f^	.02^f^	0.54
Sleep efficiency (%)	65.4 (18.6)	72 (22‐83)	66.7 (19.3)	76 (21‐82)	66.9 (19.7)	75 (18‐81)	18.7^f^ (2)	0.78^f^	.02^f^	0.92
Wake after sleep onset (h)	2.4 (1.4)	2.2 (0.2‐5)	2.3 (1.1)	2.0 (1‐4.2)	2.2 (1.4)	2.0 (0.5‐5)	20.9^f^ (2)	0.87^f^	.01^f^	0.78
Sleep regularity index (%)	72.6 (11.8)	71.3 (55‐93)	68.9 (17.3)	75.7 (44‐86)	71.2 (11.7)	69 (56‐85)	19.0^f^ (2)	0.79^f^	.01^f^	0.55
Sleep fragmentation index (%)	20.2 (13.8)	17.6 (5‐54)	26.1 (8.6)	26.5 (11‐37)	36.7 (16.2)	39.3 (12‐63)	12.4 (2)	0.51	.14	0.17
Time out of bed (min)	12.7 (4.6)	11.2 (9‐22)	12.8 (14.7)	9.4 (2.5‐48)	5.0 (2.9)	5.0 (2‐9)	12.9 (2)	0.54	.12	0.19

a No presence refers to the amount of time spent out of bed and/or out of sensor range, ICC ranges from 0 to 1, with 1 being perfect reliability or temporal stability.

b ICC: intraclass correlation coefficient.

c NPI-NH-K: Neuropsychiatric Inventory–Nursing Home–section K nighttime behaviors.

d ENMO: Euclidean norm minus one.

e milli-g: 1/1000 of the standard acceleration due to Earth’s gravity (g).

f *P*<.05, *χ*2≥5.991, Kendall *W* interpretation: 0.1‐0.29 small effect size, 0.3‐0.5 moderate effect size, >0.5 large effect size.

## Discussion

### Principal Findings

This study aimed to explore the use of sensing technologies in the assessment of longitudinal changes in physical activity levels and sleep quality in people with dementia living in a nursing home. We found that nighttime physical activity levels (ENMO) and 4 distinct digital sleep biomarkers (TST, SE, WASO, SRI) showed discrete changes across the 1-year data collection period, suggesting that these selected digital biomarkers could detect modest variations and declines in activity and sleep quality over time. Temporal stability of the sleep metrics was moderate to excellent; however, further inspection of the reliability of all longitudinal ENMO measured physical activity level data was poor. This suggests that the acceleration metric was unreliable as a longitudinal assessment tool and would be best applied to a single time-point, where reliability remained moderate to good or that for real-life use, additional sensor modalities and positions are required for better quality data. Adherence and acceptability of the selected technologies remained high across the 1-year period, with no adverse events, suggesting that the chosen devices and level of unobtrusiveness were a good match for the cohort. This study’s findings are important in the illustration of the capabilities and limitations of sensing technologies and their corresponding digital biomarkers as clinical decision-making tools for people with dementia. It also demonstrates the trade-off between the level of unobtrusiveness and quality of data when choosing sensing technologies for use within more vulnerable groups.

The standardization and clarification of measurement context for digital biomarkers is needed, as emphasized in a recent meta-analysis [48] stating that accelerometry is increasingly being used to quantify physical activity, yet daily activity metrics produced using acceleration data remain heterogeneous and primarily reflect overall movement rather than specific health-related exertion. In our study, ENMO-derived activity levels distinguished long-term group differences; however, the longitudinal reliability of these measures was poor. This is consistent with previous work [49,50] stating that the most accurate results and models occur with thigh or low back placement of sensors for the monitoring of physical activity in older adults. Therefore, device placement and user burden are central considerations; in this study’s nursing home population, a wrist-worn device was selected to minimize obtrusiveness and encourage adherence for older, potentially frail residents. While wrist-based wearables may be appropriate for single time point assessments, long-term monitoring may require alternative placements, such as the hip or low back, or integration with additional sensor modalities, to improve long-term reliability.

The World Health Organization (WHO) published the Global Action Plan on Physical Activity (GAPPA) [51,52], providing policy guidance for increased activity levels, reduced sedentary behavior, and tailored interventions across age groups. The report highlights limitations of self- and proxy-reported measures and advocates integrating wearable technologies into global physical activity surveillance. It also calls for standardized protocols to examine dose-response relationships between activity, sedentary behavior, and health outcomes, and for future research that enables data triangulation, refined estimates of total activity volume, and the identification of age- and disease-specific associations [53]. This is a promising step toward future global clinical use and recommendations for standardized metrics of accelerometry-based data; however, focused research on more vulnerable groups, such as people with dementia, is still lacking.

The landscape of sleep-monitoring technologies for individuals with dementia has evolved rapidly, integrating passive and wearable systems tailored for long-term care and home use. Traditional actigraphy remains widely used to assess sleep-wake patterns and circadian disruptions in people with dementia [48]; recently, more advanced noncontact sensors, such as under-mattress sleep mats leveraging ballistocardiograph and respiration monitoring, and radar technology, are being deployed to track heart rate, breathing, movement, and fragmentation of sleep, reducing the need for wearable devices [27,30]. Due to the rapid development of new sensing technologies that rival gold standard PSG, a recent study emphasized the importance of the evaluation of novel sleep technologies conducted within longitudinal, real-world settings including people with advanced dementia, as a pathway for future device validation [30]. These recommendations reflect a shift toward more nuanced, long-term, and minimally burdensome monitoring modalities that are critical for capturing sleep changes in nursing-home and real-world settings, while addressing the unique compliance challenges of dementia care.

The fundamental differences between traditional proxy-rated questionnaires and digital biomarkers derived from sensing technologies may capture distinct phenomena and often yield divergent conclusions [54]. The NPI-NH and PSMS are established proxy-rated instruments commonly used in nursing home research to assess behavioral symptoms, functional ability, and sleep-related disturbances. However, these questionnaires do not capture the same dimensions of sleep quality represented by the digital measures used in this study. Differences in data capture frequency also limit comparability and validation between digital and traditional tools, as traditional questionnaires are typically completed every 30 to 90 days, while sensing technologies generate continuous, real-time data. Because proxy-rated tools and sensor-based measures assess different constructs and operate across different temporal scales, they are not directly interchangeable. Future research should incorporate assessment instruments that more closely correspond to digital sleep and activity metrics to strengthen construct validity and improve clinical interpretation. We cannot, therefore, conclude that sensing technologies provide superior assessments; rather, they can offer a unique definition of physical activity levels and sleep patterns through digital biomarkers, which can strengthen and fill in the blanks between periodic traditional proxy-rated assessments.

Due to rapid development and consequential obsolescence, the future validation of sensing technologies should involve repeated measures within the same modality to accelerate validation and contextualize digital biomarker results within target populations, such as people with dementia. Global consensus on the use of a standardized metric for accelerometry data and clinical translation of the corresponding thresholds should be prioritized to improve the generalization and applicability of results in real-world settings. Establishing population and context-specific thresholds, which account for age, health status, cultural, and environmental factors, will be essential to improving generalizability, applicability, and interpretability in future studies. Until these challenges are appropriately addressed, integrating proxy-rated assessments, inclusion of human-in-the-loop interpretation for data, and cautious interpretation of digital biomarkers is most appropriate for vulnerable populations such as people with dementia residing in nursing homes.

Although this study demonstrates the use of sensing technologies and digital biomarkers, it is not without limitations. The sample size, though appropriate for a pilot study design, is small, and the results should therefore be interpreted with caution. A sensitivity analysis was conducted to account for the small sample size, the influence of extreme values, and to clarify which effects were most robust. After removing outliers and aggregating repeated measurements, the overall pattern of change remained consistent with the original results, but only WASO and TST retained statistical significance, indicating that these domains showed the strongest and most reliable longitudinal shifts. Clinically, this suggests that while participants exhibited meaningful variability across physical activity metrics and multiple sleep parameters, the most stable and reproducible changes over time occurred in wakefulness and sleep duration, whereas other measures may require larger samples to detect effects with greater confidence. Regardless, further investigation is warranted based on these preliminary results. Further, this study uses the universal metric ENMO to interpret the acceleration data into physical activity levels; however, there is currently no standardized metric established for clinical translation of physical activity digital biomarkers, and another method could produce varied results. This study’s objective was not to validate specific sensing technologies, and we would like to note that the nature of rapidly developing technologies creates a challenge for the timely validation of these novel devices and a gap between the use of these devices and digital biomarkers in research versus real-world applications.

### Conclusions

Sensing technologies are promising for supporting more objective, data-driven care models in nursing homes. Acceleration-based metrics from wrist-worn devices appear most robust for cross-sectional assessment, whereas contactless radar-derived sleep biomarkers demonstrate stronger suitability and accuracy for both snapshot and longitudinal monitoring. Our findings underscore the need for cautious, context-aware interpretation of digital biomarkers. Further work is required to establish the accuracy and reliability of these sensing methods before they are incorporated into clinical decision-making frameworks.

## Abbreviations

4AT: Assessment Test for Delirium
5-D: Decoding Death and Dying in people with Dementia by Digital thanotyping
ADL: activities of daily living
CDR: Clinical Dementia Rating
DIPH.DEM: digital phenotyping for changes in activity at the end of life in people with dementia
ENMO: Euclidean norm minus one
GAPPA: Global Action Plan on Physical Activity
GMHR: General Medical Health Rating
ICC: intraclass correlation coefficient
MAD: mean amplitude deviation
MIMS: Monitor-Independent Movement Summary
MVPA: moderate-to-vigorous physical activity
NPI-NH (K): Neuropsychiatric Inventory-Nursing home version, section K (nighttime behaviors)
PSG: polysomnography
PSMS: Physical Self Maintenance Score
REK: Regional Committee for Medical and Healthcare Research Ethics
ROCAM: Rate of Change Acceleration Movement
SE: sleep efficiency
SFI: sleep fragmentation index
SRI: sleep regulatory index
TST: total sleep time
WASO: wake after sleep onset
WHO: World Health Organization
